# Dietary Level of the Omega-3 Fatty Acids EPA and DHA Influence the Flesh Pigmentation in Atlantic Salmon

**DOI:** 10.1155/2023/5528942

**Published:** 2023-03-02

**Authors:** T. Ytrestøyl, M. Bou, C. Dimitriou, G. M. Berge, T.-K. Østbye, B. Ruyter

**Affiliations:** ^1^Nofima (Norwegian Institute of Food, Fisheries and Aquaculture Research), 6600 Sunndalsøra, Norway; ^2^Nofima (Norwegian Institute of Food, Fisheries and Aquaculture Research), 1432 Ås, Norway; ^3^Department of Animal and Aquacultural Sciences, Norwegian University of Life Sciences, Ås, Norway

## Abstract

Atlantic salmon with a start weight of 53 g were fed diets with different levels of EPA and DHA or a diet with 1 : 1 EPA+DHA (0%, 1.0%, and 2.0% of the diet). At 400 g, all fish groups were mixed and equally distributed in new tanks and fed three diets with 0.2%, 1.0%, or 1.7% of EPA+DHA. At 1200 g, the fish were transferred to seawater pens where they were fed the same three diets until they reached a slaughter size of 3.5 kg. The fillet concentration of astaxanthin and its metabolite idoxanthin was analysed before transfer to seawater pens at 1200 g and at slaughter. The fatty acid composition in the fillet was also analysed at the same time points. Salmon fed low levels of EPA and DHA had lower fillet astaxanthin concentration and higher metabolic conversion of astaxanthin to idoxanthin compared to salmon fed higher dietary levels of EPA and/or DHA. DHA had a more positive effect on fillet astaxanthin concentrations than EPA. There were positive correlations between fillet DHA, EPA, sum N-3 fatty acids, and fillet astaxanthin concentration. A negative correlation was found between the concentration of N-6 fatty acids in the fillet and the astaxanthin concentration.

## 1. Introduction

Feeds for Norwegian farmed salmon have gone through major changes in composition in the last decades, from essentially a marine based diet in the early 1990s to a diet with 70% plant ingredients today [1, 2]. One consequence of this shift in ingredients is reduced levels of the healthy N-3 fatty acids eicosapentaenoic acid (EPA) and docosahexaenoic acid (DHA) in the diet. Deficiency of these important fatty acids may have serious consequences for the salmon, and requirements for N-3 polyunsaturated fatty acids (PUFA) have been reported to range from 0.5 to 2.7% of the diet, depending on the species, life stage, and experimental conditions ([3–9]). However, it is important that studies on requirements not only assess fish growth and survival but also other important criteria such as fish health and fillet quality. The pink flesh color in salmon is important for consumer acceptance. The color intensity is determined by the deposition of keto-carotenoids such as astaxanthin in the muscle, but the basal biological mechanisms that regulate the deposition of astaxanthin in the salmon muscle have not been studied in detail. In particular, the effects of interactions between astaxanthin and other dietary components such as N-3 fatty acids, vitamin A, and other antioxidants on pigmentation are needed. Most of the existing studies are done on diet formulations with a higher content of marine ingredients than what is used in commercial farming today. Some studies report no significant effects of replacing fish oil with various plant oils on astaxanthin deposition in the muscle [10–14]. Other studies have found a positive effect of increasing the levels of N-3 PUFA in feed for Atlantic salmon on sensory-evaluated color tone [15] and fillet astaxanthin concentration [16]. Long-term studies with Atlantic salmon have shown a negative effect on flesh astaxanthin concentrations of feeding diets with low levels of N-3 fatty acids [17]. Thus, high PUFA oils may have a positive effect on astaxanthin utilization. The mechanisms are not known, and most of the studies mentioned above only measured flesh color and not digestibility or metabolism of astaxanthin, both of which may affect fillet astaxanthin deposition.

The muscle retention of astaxanthin varies with season and life stage but is typically between 5–10% whereas the digestibility of astaxanthin is often around 40% [16, 18, 19]. Thus, most of the digested astaxanthin is metabolized and/or excreted, possibly through several different pathways. Carotenoids are potent antioxidants due to their chemical structures with a long carbon chain of unconjugated double bounds quenching singlet oxygen and free radicals. Carotenoids like astaxanthin are also metabolized into diverse metabolites and cleavage products [20] that may have important biological functions of which many are linked to lipid metabolism [21]. Astaxanthin is a precursor of vitamin A in salmon and other fish [22–24], and a key gene in the regulation of this pathway in mammals is *β*,*β*-carotene-15,15′-monooxygenase (BCO1) [25, 26]. The expression of this enzyme is regulated by several dietary factors in mammals, including the content of EPA and DHA, N-6 fatty acids, vitamin A, and carotenoid content [27, 28]. Salmonids also have a reductive metabolism of ketocarotenoids [24, 29, 30]. Idoxanthin (3,3′,4′-trihydroxy-*β*,*β*-carotene-4-one) is the first metabolite in this reductive pathway of astaxanthin, and the concentration of idoxanthin and other reductive metabolites are influenced by life stage, genotype, and environmental conditions [16, 19, 30–32].

In the present study, diets with different concentrations of EPA and DHA or combinations of EPA and DHA were fed to Atlantic salmon from 53 g up to 3.5 kg, and the effects on fillet concentration of astaxanthin and its metabolite idoxanthin were measured. The fatty acid composition of the fillet was also measured at 1.2 and 3.5 kg and correlations were made between fillet astaxanthin, idoxanthin, and fatty acid compositions. A transcriptome analysis of the intestine was performed at 3.5 kg of genes related to oxidative stress and inflammation and carotenoid metabolism.

## 2. Materials and Methods

### 2.1. Experimental Design

Individually tagged (PIT-tags, passive integrated transponder; Biosonic) Atlantic salmon (S. Salar) with a mean initial weight of 52.8 g and adapted to seawater were fed two dietary levels of EPA, DHA, or a 1 : 1 mixture of EPA and DHA (1.0% and 2.0% of the diet in each dietary group) in duplicate tanks. One group was fed a diet without EPA and DHA (0%, control). This resulted in a total of 7 diets, with the same content of protein (46.6–47.0%), fat (24.6–25.9%), energy (22.1–22.6 MJ/kg), and astaxanthin (50 mg/kg) that the fish were fed until they were 400 g (period 1). The basal test diet from 52.8 to 400 g was without fish meal and fish oil but carefully formulated to meet the nutritional requirements for amino acids. EPA and DHA were added in the form of concentrates. The formulation and chemical composition of the diets and a detailed description of the experimental conditions are given in Bou et al., [3]. The fish were kept under continuous light (L:D 24 : 0) in indoor seawater tanks (1 m^3^) with flow-through seawater (33 g L^−1^ salinity). Temperature and oxygen were measured daily. The temperature varied between 6.3°C and 13.8°C (mean temperature 10.0°C), and the oxygen saturation level was over 85%. From 400 g, fish from all diet groups were evenly distributed into 9 tanks (3m^3^) with flow-through seawater on land (period 2A) and fed experimental diets containing three different levels of EPA + DHA (0.2, 1.0, and 1.7%) in triplicate until they reached approximately 1200 g. Feeds were produced as 4-, 5-, and 7-mm pellets according to fish size. The diets in the seawater period 2A in tanks on land were different in their formulation. The 0.2% EPA + DHA diet was fishmeal- and fish oil-free, and the main oil source was a mixture of poultry, rapeseed, and linseed oil (50 : 30 : 20; *v*:*v*:*v*), and the main protein source was poultry meal. The 1.0% EPA + DHA diet was also fishmeal-free, but the main source of EPA and DHA was fish oil. The composition of the 1.7% EPA+DHA diet resembled a commercial diet with respect to the content of fishmeal and fish oil. The diets contained around 32.8% fat, 36.5% protein, and 50 mg astaxanthin per kg. All diets were produced by Biomar. The feeding trials in period 1 from 50 to 400 g and period 2A from 400 to 1200 g were conducted at the Nofima Research Station in Sunndalsøra, Norway. When the fish were 1200 g, they were transported by truck to sea cages at LetSea Aquaculture Research Station in Dønna, Norway (period 2B, pens in seawater). The fish were fed diets with the same levels of EPA and DHA (0.2%, 1.0%, and 1.7%) as in tanks on land until they reached a weight of 3.5 kg. Diet concentrations of fatty acids in diets fed until 400 g (period 1), sea water period in tanks on land (period 2A), and sea cage period 2B are given in Table 1. A detailed description of diet formulation and fatty acid composition is found in Bou et al., [4]. During period 2B in sea cages, the fish were treated for sea lice 4 times (25 April, 10 July, 20 August, and 6 November) by providing chemical bath treatment with azamethiphos (0.2 mL/m^3^ for 35 min; Trident Vet, Neptune Pharma) and deltamethrin (0.3 mL/m^3^ for 30 min; ALPHA MAX, PHARMAQ). Further details on feeds and experimental conditions in seawater are found in Bou el al., [4].

### 2.2. Sampling and Analysis of Carotenoids

The effects of dietary EPA and DHA concentrations on flesh pigmentation were evaluated when the fish were 1200 g and 3.5 kg. At 1.2 and 3.5 kg, the fillet (right side) was sampled from 30 fish per diet treatment and stored on −80°C for later analysis of carotenoid and fatty acid composition as described below and in Bou et al., [3]. Fish were killed by an overdose of the anesthetic metacain (MS-222; 0.08 g/l), and individual weights and lengths were recorded. The skin and bone of the fillet were removed before the fillet was homogenized, and the carotenoids extracted using a 1: 1 : 3 mixture of distilled water, methanol (containing 500 ppm butylated hydroxytoluene), and chloroform as described by Bjerkeng et al., [33]. A Spherisorb S5CN-4800 nitrile column (Hichrom Ltd., Theale, Berkshire, UK) was used to determine the amount of astaxanthin and idoxanthin in the samples using a mobile phase with 20% (*v*/*v*) acetone in *n*-hexane+ (HPLC system I). An external standard of all-E*-*astaxanthin (Hoffmann-La Roche, Basel, Switzerland) with known concentration was prepared to establish a response line, and sample concentrations were calculated using peak areas from the chromatograms. The concentration of astaxanthin standards were determined spectrophotometrically in *n*-hexane containing 4.5% (*v*/*v*) CHCl_3_. Standards of idoxanthin 3′, 4′-*cis* and *trans* glycolic isomers were prepared according to Aas et al. [29]. The retention times (R_T_) for the 3′, 4′-*cis* and 3′, 4′-*trans* glycolic isomers of idoxanthin were ca. 7.6 and 9.3 min, respectively.

### 2.3. Fillet Colour Measurements

In instrumental tristimulus colour analysis (CIE L^∗^a^∗^b^∗^), CIE (1986) was performed on the fillets when the fish was 1200 g (30 fillets per diet treatment). The measurements were done on the dorsal muscle posterior to the dorsal fin, under the adipose fin, and in the tail on each fillet, using a Minolta Chroma Meter CR-300 (Minolta, Osaka, Japan). Measurements were made directly on the fillets, and the measuring head was rotated 90^o^ between duplicate measurements per position, and means of six recordings per fish were used for data analysis. Visual color assessment using a Roche SalmoFan (Hoffmann-La Roche, Basel, Switzerland) was done on of the same fillets at the same positions. The color was evaluated using a scale between 20 and 34 (20 represents a low degree of pigmentation and 34 represents a highly pigmented fillet).

### 2.4. RNA Isolation, cDNA Synthesis, and Quantification of Transcript Levels by Real-Time Quantitative PCR

Total RNA was isolated from the intestine at the final sampling (3.5 kg) using a PureLink Pro 96 RNA Purification Kit (Invitrogen), according to the manufacturer's instructions. RNA was treated with PureLink DNaseI (Invitrogen) to remove any contaminating DNA. RNA concentration was measured using a NanoDrop® ND-1000 spectrophotometer (NanoDrop Technologies). Reverse transcription of 1 *μ*g total RNA into complementary DNA (cDNA) was carried out using a TaqMan® Reverse Transcription Reagents kit (Applied Biosystems) according to the manufacturer's protocol in a 20-*μ*l reaction volume.

PCR primers (Table 2) were designed using Vector NTI (Invitrogen) and synthesized by Invitrogen. The efficiency was checked from 10-fold serial dilutions of cDNA for each primer pair. Real-time PCR was performed in a LightCycler 480 Instrument (Roche Applied Science). The PCR master mix consisted of 0.5 *μ*l forward and 0.5 *μ*l reverse primer (0.5 *μ*M final concentrations), 4 *μ*l of a 1 : 10 dilution of cDNA and 5 *μ*l LightCycler 480 SYBR® Green I Master (Roche Applied Science). All samples were analysed in duplicate with a non-template control for each gene. The reaction was performed by incubating the samples at 95°C for 5 min, forty-five cycles of 95°C for 15 s and 60°C for 15 s, and 72°C for 15 s for denaturation, annealing, and extension, respectively. The specificity of PCR amplification was confirmed by melting curve analysis (95°C for 5 s and 65°C for 1 min, and a continuous temperature ramp (0.11°C/s) from 65 to 97°C). Eukaryotic translation initiation factor 3 (*etif3*), RNA polymerase 2 (*rpol2*), and elongation factor 1-alpha (*ef1a*) were evaluated as reference genes, and it was found that the latter was the most stable. Relative expression levels of mRNA transcripts were calculated using the -*ΔΔ*Ct method using *ef1a* as the reference gene [34].

### 2.5. Statistical Analysis

Diet effects on fillet astaxanthin and idoxanthin concentration and color were analysed by ANOVA in SAS jmp with diet in period 1 (50–400 g) and period 2 (400–3500 g) as fixed factors. *P* values <0.05 were considered significant differences and *P* < 0.1 were considered a trend. Linear correlations were made between fillet content of astaxanthin, idoxanthin, content of EPA, DHA, sum N-3, N-6, and saturated (N-0) fatty acids. Correlation analyses were done in SAS jmp. Principal component analysis (PCA) was performed using muscle concentrations of astaxanthin, idoxanthin, and fatty acid composition (content of EPA, DHA, sum N-0, N-3, N-6, and N-9) as input variables using the software Unscrambler® X, version 10.3 (CAMO).

## 3. Results

### 3.1. Fatty Acid Composition of the Fillet

The fatty acid composition of the fillets at 1.2 and 3.5 kg are shown in Tables 3 and 4, respectively. The fatty acid composition of the fillet reflected the dietary fatty acid composition and showed a 2.2-fold increase in percentage of EPA, a 1.7-fold increase in percentage of DHA and a 1.6-fold decrease in percentage of N-6 fatty acids as EPA + DHA in the diets increased from 0.2% to 1.7% in the diets. When looking into prediet effects in these two sizes of fish, fish fed increasing dietary levels of EPA and DHA in period 1 had significantly higher muscle levels of these fatty acids when they reached 1.2 kg (Table S1), although they had been fed the same diets since they were 400 g. The effects of the prediets were still significantly present at the final sampling, when the fish reached 3.5 kg, with fish fed higher content of EPA+DHA during the early life stage showing higher levels of these FA regardless of the main diet received during period 2 (Table S2).

### 3.2. Carotenoid Concentrations and Fillet Color

**Figure 1:**
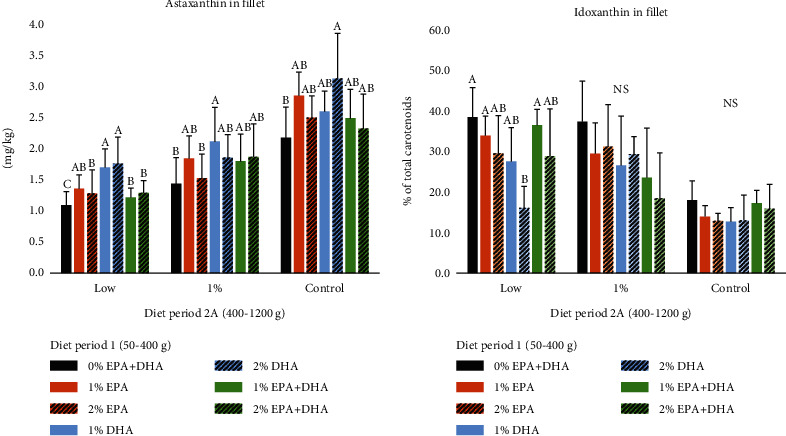
Fillet astaxanthin (a) and idoxanthin (b) in 1200 g salmon grouped by diet in period 1 (7 diets from 50 to 400 g containing 0, 1, and 2% EPA and DHA, and EPA and DHA in combination) and by diet in period 2A (3 diets from 400-1200 g) containing 1.7% EPA + DHA (Control), 1% EPA + DHA, and 0.2% EPA+DHA (low). Values are mean per treatment ± SEM. *n* = 3 tanks per treatment. Significant differences between diet groups in period 1 are indicated by different letters (*P* < 0.05).

**Figure 2:**
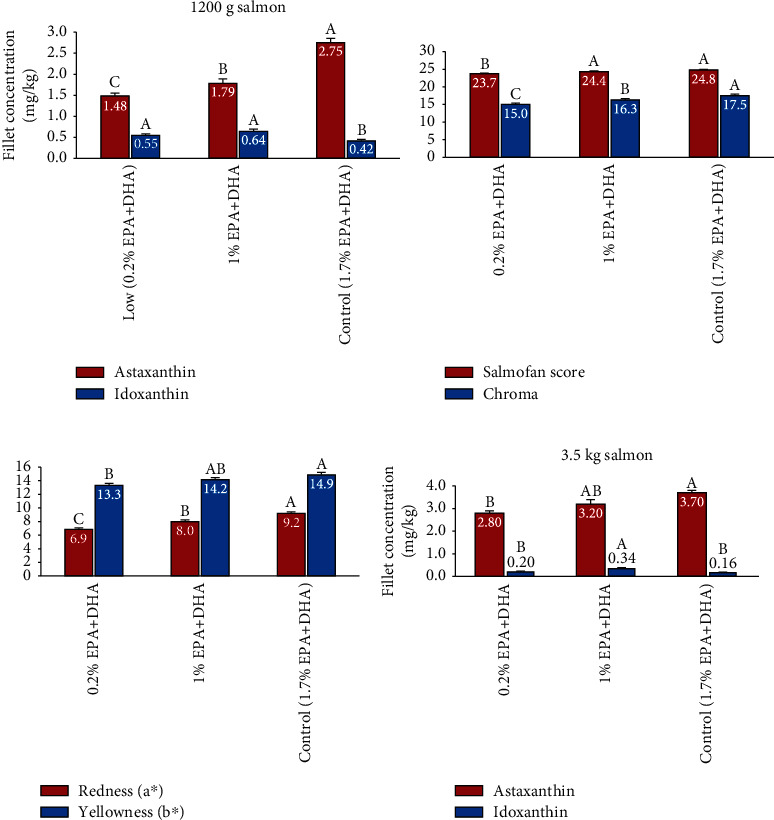
Visual color and concentration of astaxanthin and idoxanthin in salmon fillets at 1200 g. Data from fish fed, the 7 different diets in period 1 (50–400 g) are pooled. (a) fillet astaxanthin and idoxanthin, (b) Salmofan and chroma of fillets, c) Minolta redness (a^∗^) and yellowness (b^∗^), and (d) Fillet concentration of astaxanthin and idoxanthin at 3.5 kg. Values are means per treatment (diet period 2) ± SEM. Means labelled with common letters are not significantly different (*P* < 0.05).

When the salmon was 1200 g, the major effects on fillet astaxanthin and idoxanthin concentrations were due to the diet fed during period 2B (400–1200 g) (*P* < 0.0001, Figure 1). However, there were also some significant effects of the diet used in period 1 (50–400 g) on muscle carotenoid concentration (*P* < 0.05). Fish that had been fed a diet without EPA and DHA from 50 to 400 g (0%) had lower astaxanthin concentration in the fillet, compared to fish fed diets containing EPA and DHA (Figure 1(a)). Fish fed diets with DHA in period 1 had the highest astaxanthin concentrations. There was no significant effect of dietary content of EPA and DHA in period 1 on fillet idoxanthin concentration at 1200 g in fish fed 1% EPA+DHA or 1.7% EPA+DHA in period 2B (Figure 1(b)). In salmon fed diets with low EPA and DHA concentration in period 2 there was a tendency for higher idoxanthin concentrations in fillets of salmon fed low levels of EPA and DHA in period 1 (*P* = 0.09, Figure 1(b)). However, the major effect on fillet astaxanthin concentration at 1200 g was the diet concentration of EPA and DHA in period 2B (400–1200 g). Salmon fed the diet with 0.2% EPA+DHA (low) had the lowest fillet astaxanthin concentration with a mean of 1.38 mg/kg when all diet groups from period 1 are pooled (Figure 2(a)). Salmon fed the 1.0% and the 1.7% diets in period 2A had 1.79 and 2.75 mg astaxanthin per kg, respectively, when all diets in period 1 were pooled. The differences in fillet astaxanthin were also reflected in fillet visual color measured by SalmoFan scores and Minolta chroma, its redness and yellowness (Figures 2(b) and 2(c)). Fillets from salmon fed diets containing 0.2% EPA and DHA from 400 to 1200 g were less colored than fillets from fish fed the diets containing 1.0% or 1.7% EPA and DHA (*P* < 0.0001). However, the diet provided to the fish during period 1 (50–400 g) did not have a significant effect on fillet color of 1200 g salmon measured as Salmofan score, chroma, redness, and yellowness assessed by Minolta. There was a significant effect of diet in period 2A on the concentration of idoxanthin in the fillet (*P* < 0.0001). Salmon fed the commercial control diet (1.7% EPA+DHA) had lower idoxanthin concentration compared to salmon fed the 0.2 and 1.0% diets (Figure 2(a)). Idoxanthin amounted 14% of the total carotenoid content in fillet of salmon fed the 1.7% control diet (all diets in period 1 pooled) whereas the idoxanthin concentration was around 30% of total carotenoids in the 0.2 and 1.0% diets (pooled mean for all diets period 1). There were significant negative correlations between fillet concentrations of EPA, DHA, sum N-9 fatty acids, and % idoxanthin of total carotenoids (*P* < 0.0001, *R*^2^ = 0.27, 0.25 and 0.25, respectively).

**Figure 3:**
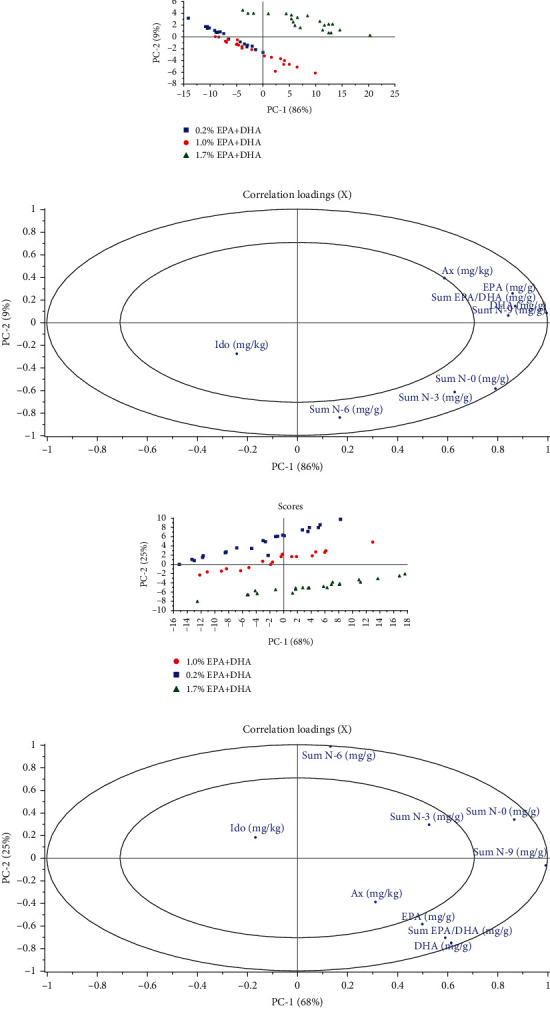
PCA plots Scores (A, C) and correlation loadings (B, D) showing the relationship between the muscle samples and their FA, astaxanthin, and idoxanthin content. Plots A and B show the first principal component (PC-1) compared with the second principal component (PC-2), summarizing 95% of the variation between diet groups from 1.2 kg salmon. Plots C and D show PC-1 compared with PC-2, summarizing 93% of the variation between diet groups from 3.5 kg salmon.

Correlations between fillet astaxanthin and fatty acid composition showed a positive correlation between fillet DHA and EPA concentration and astaxanthin concentration (*P* < 0.0001, *R*^2^ = 0.43 and 0.46, respectively) and a negative correlation with N-6 fatty acids (*P* < 0.01, *R*^2^ = 0.13). PCA analysis showed no clear pattern related to diet in period 1. The diet in period 2 was responsible for the differences observed (Figure 3).

There was no significant effect of diet in period 1 on fish bodyweight at 400 g. There was however an effect of diet in period 2A on the bodyweight; the fish fed the commercial control diet with a 1.7% of EPA+DHA were 1.3 kg; the fish fed with a1% of EPA+DHA diet were 1.2 kg; and the fish fed with a low EPA + DHA diet were 1.1 kg when the fish were transferred to pens in seawater (*P* < 0.0001). Linear regressions between fish weight and fillet astaxanthin and idoxanthin at 1200 g were made to test whether fish weight had an effect on fillet color. For the 0.2 and 1.0% diet, there was no significant relationship between fish weight and fillet color, astaxanthin, or idoxanthin concentration. For the commercial control diet (1.7% EPA+DHA) there was a significant positive correlation between bodyweight and fillet astaxanthin, chroma, redness and yellowness (*P* < 0.01), with bodyweight explaining 9% of the total variation in fillet colour parameters and 12% of the variation in fillet astaxanthin concentration.

There were no significant differences in bodyweight between diets at the final sampling when the salmon weighed around 3.5 kg. There was however a clear positive effect of a higher dietary concentration of omega-3 fatty acids on fillet astaxanthin concentration. The effect of diet in period 1 on fillet astaxanthin or idoxanthin concentrations was no longer significant. Data from the dietary groups in period 1 is therefore pooled in Figure 2(d). The fillet astaxanthin concentration was significantly affected by diet fatty acid composition in period 2 A and B, between 400 and 3.5 kg (*P* < 0.001). Salmon fed the 0.2% diet had the lowest fillet astaxanthin concentration and fish fed the commercial control diet with a 1.7% EPA+DHA had the highest fillet astaxanthin concentration. The concentration of idoxanthin in the fillet at 3.5 kg was also affected by diet (Figure 2(d)). As in the 1200 g fish, it was the highest in the 1% diet and lowest in the commercial control diet (*P* < 0.001). There was a significant linear correlation between fillet content of EPA and DHA (*P* < 0.0001, *y* = 0.81*x* + 0.75, *R*^2^ = 0.64). Positive correlations between fillet concentrations of astaxanthin and content of EPA and DHA were found (*P* < 0.0001) explaining around 25% of the variation in fillet astaxanthin. There were also positive correlations between sum of N-3 and N-0 fatty acids and fillet astaxanthin concentration (*P* < 0.0001, *R*^2^ = 0.26 and 0.28, respectively) whereas a weaker negative correlation with sum N-6 fatty acids in fillet was found (*P* < 0.001, *R*^2^ = 0.11). For idoxanthin in fillet (% of total carotenoids) weak negative correlations were found with concentration of DHA in fillet (*P* < 0.05, *R*^2^ = 0.06) and sum N-0 in fillet (P < 0.01, *R*^2^ = 0.09).

### 3.3. Gene Expression in Intestine

**Figure 4:**
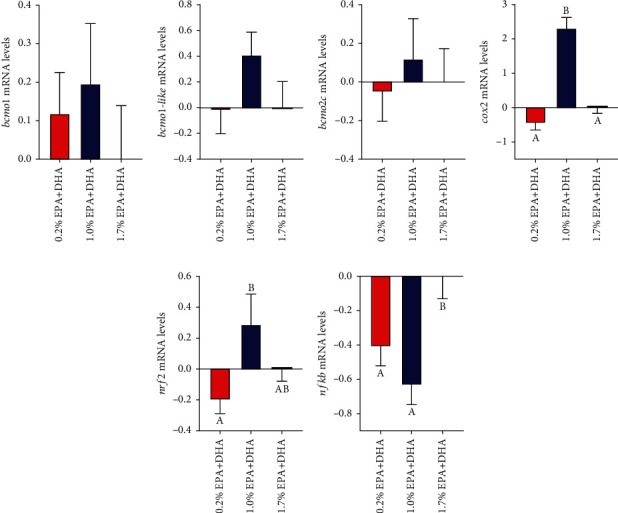
Expression of the genes bcmo1 (a), bcmo2c (b), bcmo1-like (c), cyclooxygenase 2 (d) NF-E2-related factor 2 (e), nuclear factor-kappa B (f) in the mid-intestine in salmon fed diets containing 1.7% EPA+DHA (control), 1% EPA+DHA, and 0.2% EPA+DHA (low) from 400–3500 g (period 2). Data from fish fed different diets in period 1 (40–400 g) are pooled. Values are means per treatment (diet period 2) ± SEM. Means labelled with common letters are not significantly different (*P <0.05*).

The expression of the *β*-carotene oxygenases bmco1, bmco1-like, and bmco2-c in the intestine was measured at the final sampling at 3.5 kg. There were no significant effects of diet in periods 1 or 2 on the expression on the three carotenoid oxygenase genes (Table 5). The diet treatments in period 1 is therefore not shown, but instead pooled within diet treatment in period 2 (Figures 4(a)–4(c)). Overall, salmon fed 1% shoved the numerically the highest expression of all three genes in the intestine, but the differences were not statistically significant due to large variation between individuals. At the final sampling, expression of genes involved in cellular defense mechanisms against oxidative stress and inflammation were also measured in the intestine. The expression of cyclooxygenase 2 (*cox2*), a gene involved in synthesis of proinflammatory eicosanoids, was elevated in fish fed the 1% diet compared to the two other diets (Figure 4(d)). There was also an effect of diet from 50 to 400 g, where increasing levels of EPA and DHA in early life stages down regulated the expression of *cox2* (Figure S1). The expression of the gene coding for nuclear factor erythroid 2-related factor 2 (*nrf-2*) involved in regulation of genes protecting against oxidative damage was upregulated in fish fed 1.0% EPA+DHA compared to in salmon fed diets with a 0.2 and 1.7% of EPA+DHA (Figure 4(e)). Reduced expression of the transcription factor NF-*κ*B was found in diets with a 0.2 and 1.0% EPA + DHA compared to the salmon fed with a 1.7% diet (Figure 4(f)).

## 4. Discussion

The reduction in dietary concentration of the important omega-3 fatty acids EPA and DHA in later years due to the replacement of marine ingredients with plant ingredients may have consequences for salmon health and quality. Requirements have often been assessed based on growth effects in trials of short duration under experimental conditions without stress, and many of the trials have been done on juveniles in freshwater. However, requirements cannot be based only on growth effects, but also have to consider fish health, welfare, and quality [3, 4]. Requirements for large salmon under challenging conditions in seawater are also needed to ensure fish health and the quality of the final product. Bou et al. [4] found that 1% EPA+DHA in the Atlantic salmon diet, a level previously regarded as sufficient, was too low to maintain fish health under demanding environmental conditions in sea cages. Low EPA+DHA resulted in increased mortality during delousing at high water temperatures, increased fat content in the liver, intestine, and viscera, reduced intervertebral space, and caused mid-intestinal hyper-vacuolization. EPA and DHA in the different tissue membrane phospholipids were typically replaced by pro-inflammatory N-6 fatty acids. In the present study, negative effects of low levels of EPA and DHA on flesh pigmentation and a positive correlation between muscle concentrations of astaxanthin and N-3 fatty acids were found, whereas a negative correlation between astaxanthin concentration and muscle content of N-6 fatty acids was observed. Long chain omega-3 fatty acids may improve pigmentation through several mechanisms. Fatty acid composition affects the solubility and transfer of carotenoids into the aqueous phase of carotenoids in triglyceride emulsions [35]. Differences in the transfer efficiency of astaxanthin into intestinal micelles may explain the effects of some dietary oils observed on astaxanthin deposition. Saturated fatty acids have been shown to be negative for the digestibility of astaxanthin at low temperatures [36], but not at higher temperatures. Studies on interactions between PUFAs and temperature on astaxanthin digestibility *in vivo* are however, lacking in salmon, and the digestibility was not measured in the present study.

In the present study, the diet raw material composition was identical until the fish were 400 g, but from 400 g until 3.5 kg, the diets differed in the content of fish oil and fish meal [4]. The effects seen in diet in period 1 can thus be attributed to the differences in EPA and DHA concentration, but in period 2, the effects could also potentially be a result of the different fatty acid composition, in particular of the N-6 fatty acid content which was higher in the 0.2% diet and lowest in the 1.7% diet [4]. The fatty acid concentration of the fillet reflected that of the diet, but the fatty acid concentration at slaughter was still significantly affected by diet EPA and DHA content in Period 1 from 50 to 400 g. Although there was no longer a significant effect of diet in period 1 on fillet astaxanthin at slaughter, a stronger positive correlation between fillet astaxanthin and EPA and DHA content was found at slaughter than the negative correlation found between astaxanthin and N-6 fatty acids in the fillet at slaughter. Some other studies support the findings in the present study. In a study on Atlantic salmon, Waagbø et al. [17] replaced 64% of the dietary fish oil with vegetable oil and reduced the fishmeal content in a full-scale seawater production. Reduced growth and feed efficiency were observed with decreasing fishmeal inclusion levels. When the low marine diets were boosted with a South American omega-3 fish oil three months prior to slaughter, fillet color and astaxanthin content improved significantly. The average astaxanthin concentration was 5.0 mg kg^−1^ in Norwegian Quality Cut (NQC) samples from all fish groups taken after one year of feeding, with no difference between the dietary groups. After an additional 5 months, the average astaxanthin content in NQC samples was still 5.5 mg kg^−1^ for fish groups fed the control low-marine diets, but 6.2 mg kg^−1^ in the fish groups fed the omega-3- boosting diets. However, there were no significant differences between diets in visual color measured by SalmoFan and Minolta. Some earlier studies have also shown positive effects of high-PUFA oils on flesh pigmentation. Atlantic salmon fed diets with high Peruvian PUFA oil deposited 13% more carotenoids in the fillet than fish fed diets supplemented with herring oil, and a positive linear relationship was found between final fillet idoxanthin concentration and total saturated fatty acids in supplement oils [16]. Fillets of Atlantic salmon fed diets containing 29% Peruvian, high-PUFA fish oil contained 41% more carotenoids (canthaxanthin and astaxanthin combined) than fillets of salmon fed a diet with 29% soybean oil ([37, 38]). Since most of the studies that have examined the effect of diet fatty acid composition on fillet color have replaced fish oils or fish meal with different plant oils and protein sources, it is not possible to say if the effect on pigmentation is due to a higher content of PUFA in the diet or a lower content of N-6 fatty acids.

The N-6 fatty acids are known to promote the formation of pro-inflammatory and pro-aggregatory eicosanoids, whereas the N-3 fatty acids have the opposite effects. Carotenoids such as astaxanthin are potent antioxidants due to their chemical structure with a long carbon chain of unconjugated double bounds quenching singlet oxygen and free radicals [21, 39]. Higher oxidative stress levels in the salmon could thus result in more astaxanthin being oxidized in the salmon and lead to reduced astaxanthin levels in the muscle. Low EPA+DHA levels also increased fat deposition in the liver, intestine, and viscera and caused mid-intestinal hyper-vacuolization in the 0.2 and 1% EPA+DHA groups [4]. There were also indications of a higher oxidative stress level in the intestine of salmon fed the 1.0% diet. The transcription factor nrf2 (NF-E2-related factor 2) is a potent transcriptional activator and plays a central role in inducible expression of many cytoprotective genes in response to oxidative stress [40, 41]. The expression of *nrf-2* in the intestine was higher in fish fed 1.0% EPA+DHA than in fish fed 0.2% EPA+DHA, while in fish fed, the control diet had intermediate expression. Salmon fed the diet with a 1% of EPA+DHA also had the highest expression of cyclooxygenase 2 (*cox2*), a gene involved in the synthesis of proinflammatory eicosanoids. The nuclear factor-kappa B (NF-*κ*B) is a transcription factor that plays a key role in the control of genes involved in inflammation, cell proliferation, and apoptosis and is activated in response to inflammatory stimuli and environmental stressors [42]. Reduced expression of *nf-κb* was found in the intestine of salmon fed diets with 0.2 and 1.0% EPA+DHA compared to the control diet with 1.7% EPA and DHA which could suggest less response in these groups. However, the regulation of expression *nf-κb* in mammals is shown to be quite complex and also mediated through feed-back regulation [43]. Little information is available on the regulation of this pathway in salmon, but a negative feedback mechanism could explain lower expression of this gene in salmon fed diets with lower levels of EPA and DHA.

Carotenoids are transformed into diverse metabolites and cleavage products that may have important biological functions including regulation of expression of genes involved in cell metabolism and defense against oxidative damage [20, 25]. In mammals, carotenoids have been shown to lower blood concentrations of inflammation markers and pro-inflammatory cytokines, improve insulin sensitivity, and reduce obesity [44, 45]. Many of these biological effects of carotenoids in mammals are linked to the activity of carotenoid oxygenases that cleaves *β*-carotene as the first step in retinol synthesis [26]. Two distinct forms of this enzyme are found in mammals: beta-carotene 15,15′-monooxygenase (BCMO1), and beta-carotene-9,10-dioxygenase 2 (BCDO2). The Atlantic salmon beta-carotene oxygenase gene family contains 5 members, three *bco2*, and two *bcmo1* paralogs (*bcmo1* and *bcmo1 like*) that have tissue-specific expression, with the highest expression in the liver and intestine and lowest in the muscle [46]. The regulation of the carotenoid oxygenases has been studied in mammals, but little is so far known for fish. The presence of a PPAR*γ* response element in the upstream of BCMO1 promoter, may explain the connection between carotenoid and lipid metabolism in mammals [47], but if this is also the case in salmon is not known. Dietary factors, including type and amount of dietary fat have been shown to influence the activity of BCMO1 in mammals [28]. Unsaturated N-3 fatty acids were shown to enhance *β*,*β*-carotene 15,15′-dioxygenase activity in rat intestine [27, 48]. However, in the present study, there were not found any clear effects of dietary content of N-3 fatty acids on gene expression of carotenoid oxidases in the intestine. Overall, the 1% diet shoved the highest expression of all three genes, but the differences were not statistically significant due to the large variation between individuals.

## 5. Conclusions

Low levels of EPA and DHA in the diet were negative for salmon flesh pigmentation, both in a tank environment and in seawater pens. The fillet concentration in all treatments was quite low for a salmon of 3.5 kg. The fish in this trial had several delousing operations during the seawater period that led to elevated mortality when the water temperature was high. There is currently little information on how these essential nutrients interact and how stress may affect the dietary requirements necessary for optimal health and quality of the salmon. Handling in connection with delousing have become more frequent in commercial salmon farming in recent years, and it has also become more challenging to obtain the sufficient flesh color. Although not providing a casual explanation, the present study shows a connection between low dietary EPA and DHA levels and reduced flesh color in salmon.

## Figures and Tables

**(a):** 

Period 1 (50–400 g)	Control (0%)	1% EPA	2% EPA	1% DHA	2% DHA	1% EPA+DHA	2% EPA+DHA
Total lipid	25.2	25.1	25.4	24.6	25.7	25.4	25.9
∑ SFA	21.2	19.8	18.1	20.1	19.2	20.0	18.8
∑ MUFA	48.9	45.1	41.9	45.8	43.5	46.1	42.6
∑ PUFA	29.1	33.3	36.5	32.2	35.9	32.6	36.4
EPA	0.1	4.3	8.3	0.7	1.4	2.6	5.1
DHA	0.1	1.2	2.1	4.0	8.1	2.6	5.1

**(b):** 

	2 g/kg EPA+DHA	10 g/kg EPA+DHA	17 g/kg EPA+DHA
Period 2A (400-1200 g, tanks with seawater)			
Total lipid	31.1	30.7	28.8
∑ SFA	20.7	22.9	14.2
∑ MUFA	43.4	40.3	52.5
∑ PUFA	35.6	36.4	32.4
EPA	0.2	2.2	3.5
DHA	0.2	1.9	3.0
Period 2B (1200–3500 g, pens in seawater)			
Total lipid	32.4	34.5	31.6
∑ SFA	20.8	19.3	15.1
∑ MUFA	44.1	49.8	60.8
∑ PUFA	34.2	29.5	20.1
EPA	0.4	2.4	3.6
DHA	0.3	1.1	2.1

12 : 0, 15 : 0, 17 : 0, and 24 : 0. 14 : 1 n-5, 15 : 1, 16 : 1 n-5, 16 : 1 n-9, 17 : 1 n-7, 20 : 1 n-7, and 22 : 1 n–7. 20 : 3 n-6. ^4^18 : 3 n-4.

**Table 2:** Atlantic salmon primer sequences used for real-time PCR.

Gene	Accession no.	Direction	Primer sequence 5′⟶3′
ef1*α*	AF321836	Forward	CACCACCGGCCATCTGATCTACAA
		Reverse	TCAGCAGCCTCCTTCTGAACTTC

etif3	DW542195	Forward	CAGGATGTTGTTGCTGGATGGG
		Reverse	ACCCAACTGGGCAGGTCAAGA

rpol2	CA049789	Forward	TAACGCCTGCCTCTTCACGTTGA
		Reverse	ATGAGGGACCTTGTAGCCAGCAA

bcmo1	NM001279071	Forward	TGTTGCAATGTCAGCAGTGG
		Reverse	AATGAACACGGGCTCTGATG

bcmo1-like	NM001279071	Forward	ATGGCTCAAGAGTGGAGTGG
		Reverse	GGCTCAGAGGGGTAGCAGTC

bcmo2c		Forward	GACACCTGTTCAGCGACTCC
		Reverse	CAGGCTCAGATGGGAACAGA

cox2	AY848944	Forward	CCCCCGACTTACAATGCTGA
		Reverse	GCGGTTCCCATAGGTGTAGG

Nfkb	CA341859	Forward	CAGCGTCCTACCAGGCTAAAGAGAT
		Reverse	GCTGTTCGATCCATCCGCACTAT

nrf2	BT059007	Forward	CCGGACTCCTCGCCTTCGGA
		Reverse	GTGGATAGTTGGCTTGTCCCTTCGT

*ef1 α*: elongation factor 1A;.*etif3*: eukaryotic translation initiation factor 3; *rpol2*: RNA polymerase 2; *bcmo1*: beta-carotene 15,15′-monooxygenase 1; *bcmo1-*like: beta-carotene 15,15′-monooxygenase 1 like; *bco2c*: beta-carotene oxygenase 2;*cox2:* cyclooxygenase 2; *nfkb*: nuclear factor kappa B1; *nrf2*: nuclear factor erythroid 2-related factor 2.

**Table 3:** Fatty acid composition (% of total) in the muscle of Atlantic salmon (1200 g) fed the experimental diets from 400 g. Data are shown as mean ± SEM (*n* = 24, being each sample represented by a pool of 2 fish).

	0.2% EPA+DHA	1.0% EPA+DHA	1.7% EPA+DHA	ANOVA
Total fat	8.3 ± 0.17^c^	9.5 ± 0.18^b^	10.5 ± 0.26^a^	<0.0001
14 : 0	0.8 ± 0.01^c^	1.9 ± 0.02^b^	2.9 ± 0.04^a^	<0.0001
16 : 0	11.7 ± 0.92^ab^	13.2 ± 0.06^a^	11.0 ± 0.03^b^	0.0183
18 : 0	4.4 ± 0.02^a^	4.0 ± 0.02^b^	2.7 ± 0.02^c^	<0.0001
*Σ* n-0	18.0 ± 0.92^b^	20.2 ± 0.07^a^	17.6 ± 0.05^b^	0.0032
16 : 1 n-7	2.2 ± 0.01^c^	3.4 ± 0.03^b^	4.1 ± 0.05^a^	<0.0001
18 : 1 n-9	37.3 ± 0.11^a^	32.0 ± 0.28^c^	33.4 ± 0.40^b^	<0.0001
20 : 1 n-9	1.3 ± 0.01^c^	3.3 ± 0.15^b^	6.9 ± 0.14^a^	<0.0001
22 : 1 n-9	0.2 ± 0.00^c^	0.5 ± 0.01^b^	4.1 ± 0.50^a^	<0.0001
*Σ* MUFA	45.3 ± 0.92^b^	41.6 ± 0.31^c^	50.4 ± 0.26^a^	<0.0001
18 : 2 n-6	17.9 ± 0.06^a^	14.6 ± 0.09^b^	11.3 ± 0.09^c^	<0.0001
18 : 3 n-6	0.8 ± 0.02^a^	0.3 ± 0.02^b^	0.3 ± 0.01^c^	<0.0001
20 : 4 n-6	1.2 ± 0.02^a^	0.6 ± 0.01^b^	0.4 ± 0.01^c^	<0.0001
*Σ* n-6	20.9 ± 0.05^a^	16.6 ± 0.08^b^	12.9 ± 0.10^c^	<0.0001
18 : 3 n-3	7.7 ± 0.04^b^	8.5 ± 0.04^a^	3.8 ± 0.03^c^	<0.0001
20 : 5 n-3	1.2 ± 0.05^c^	1.9 ± 0.05^b^	2.6 ± 0.03^a^	<0.0001
22 : 5 n-3	0.4 ± 0.01^c^	0.6 ± 0.01^b^	1.0 ± 0.01^a^	<0.0001
22 : 6 n-3	2.6 ± 0.07^c^	3.9 ± 0.07^b^	4.4 ± 0.09^a^	<0.0001
*Σ* n-3	12.0 ± 0.10^b^	15.2 ± 0.11^a^	12.0 ± 0.14^b^	<0.0001

**Table 4:** Fatty acid composition (% of total) in the muscle at slaughter (3.5 kg) of Atlantic salmon fed the experimental diets for 26 weeks (period 1) and period 2 A and B (13 months). Data are shown as mean ± sem (*n* = 24, being each sample represented by a pool of 2 fish).

	0.2% EPA+DHA	1.0% EPA+DHA	1.7% EPA+DHA	ANOVA
Total fat	15.3 ± 0.31^b^	16.3 ± 0.48^b^	17.8 ± 0.43^a^	<0.0001
14 : 0	1.2 ± 0.04^c^	2.6 ± 0.03^b^	3.7 ± 0.02^a^	<0.0001
16 : 0	12.6 ± 0.07^a^	11.9 ± 0.07^b^	9.9 ± 0.03^c^	<0.0001
18 : 0	4.2 ± 0.04^a^	3.4 ± 0.02^b^	2.2 ± 0.02^c^	<0.0001
*Σ* n-0	18.5 ± 0.08^a^	18.4 ± 0.15^a^	16.4 ± 0.06^b^	<0.0001
16 : 1 n-7	2.8 ± 0.05^c^	4.1 ± 0.04^b^	4.9 ± 0.03^a^	<0.0001
18 : 1 n-9	37.5 ± 0.22^a^	30.1 ± 0.18^b^	29.6 ± 0.09^b^	<0.0001
20 : 1 n-9	1.9 ± 0.11^c^	5.6 ± 0.07^b^	9.3 ± 0.06^a^	<0.0001
22 : 1 n-9	0.2 ± 0.03^b^	0.7 ± 0.09^a^	0.8 ± 0.19^a^	0.005
*Σ* MUFA	48.0 ± 0.14c	51.1 ± 0.68^b^	60.0 ± 0.16^a^	<0.0001
18 : 2 n-6	17.7 ± 0.13^a^	13.1 ± 0.10^b^	9.7 ± 0.04^c^	<0.0001
18 : 3 n-6	0.6 ± 0.04^a^	0.3 ± 0.01^b^	0.2 ± 0.01^c^	<0.0001
20 : 4 n-6	0.4 ± 0.01^a^	0.3 ± 0.01^b^	0.2 ± 0.00^c^	<0.0001
*Σ* n-6	20.7 ± 0.17^a^	15.3 ± 0.23^b^	11.4 ± 0.05^c^	<0.0001
18 : 3 n-3	8.1 ± 0.04^a^	8.2 ± 0.05^a^	3.5 ± 0.01^b^	<0.0001
20 : 5 n-3	1.4 ± 0.05^c^	2.1 ± 0.07^b^	2.6 ± 0.14^a^	<0.0001
22 : 5 n-3	0.4 ± 0.02^c^	0.7 ± 0.02^b^	1.0 ± 0.01^a^	<0.0001
22 : 6 n-3	1.4 ± 0.05^c^	2.3 ± 0.07^b^	3.0 ± 0.04^a^	<0.0001
*Σ* n-3	11.8 ± 0.08^b^	14.0 ± 0.30^a^	10.5 ± 0.14^c^	<0.0001

**Table 5:** *P* values for effects of diet in periods 1 or 2 on the expression on the three carotenoid oxygenase genes and *cox*2, *nrf*2 and *nfkb.*

	*P*_Diet period 2_	*P*_Diet period 1_	*P*_Diet period 1×diet period 2_
*bcmo1*	0.4325	0.7098	0.7552
*bcmo1-like*	0.2496	0.5866	0.1802
*bcmo2c*	0.7046	0.5563	0.1414
*cox2*	<0.0001	<0.05	0.0584
*nrf2*	0.1105	0.7922	0.5777
*Nfkb*	<0.05	0.4503	0.5801

## Data Availability

The data used to support the findings of this study are included within the article.

## Supplementary Materials

Supplementary Materials The tables in the supplemental files show the muscle fatty acid composition at 1.2 (Table S1) and 3.5 kg (Table S2) in all dietary treatments. Figure S1 shows the effect of diet in Period 1 (50-400 g) and Period 2 (400-3500 g) on the expression of the *cox*2 gene when the fish was 3.5 kg.

## References

[1] Aas T. S., Åsgård T., Ytrestøyl T. (2022). Utilization of feed resources in the production of rainbow trout (*Oncorhynchus mykiss*) in Norway in 2020. *Aquaculture Reports*.

[2] Ytrestøyl T., Aas T. S., Åsgård T. (2015). Utilisation of feed resources in production of Atlantic salmon (*Salmo salar*) in Norway. *Aquaculture*.

[3] Bou M., Berge G. M., Baeverfjord G. (2017). Requirements of n-3 very long-chain PUFA in Atlantic salmon (*Salmo salar* L): effects of different dietary levels of EPA and DHA on fish performance and tissue composition and integrity. *The British Journal of Nutrition*.

[4] Bou M., Berge G. M., Baeverfjord G., Sigholt T., Østbye T.-K., Ruyter B. (2017). Low levels of very-long-chain n-3 PUFA in Atlantic salmon (*Salmo salar*) diet reduce fish robustness under challenging conditions in sea cages. *Journal of Nutritional Science*.

[5] Glencross B. D. (2009). Exploring the nutritional demand for essential fatty acids by aquaculture species. *Reviews in Aquaculture*.

[6] Lutfi E., Berge G. M., Bæverfjord G. (2023). Increasing dietary levels of the omega-3 long-chain polyunsaturated fatty acids, EPA and DHA, improves the growth, welfare, robustness, and fillet quality of Atlantic salmon in sea cages. *British Journal of Nutrition*.

[7] Qian C., Hart B., Colombo S. M. (2020). Re-evaluating the dietary requirement of EPA and DHA for Atlantic salmon in freshwater. *Aquaculture*.

[8] Rosenlund G., Torstensen B., Stubhaug I., Usman N., Sissener N. (2016). Atlantic salmon require long-chain *n*-3 fatty acids for optimal growth throughout the seawater period. *Journal of Nutritional Science*.

[9] Ruyter B., Røsjø C., Einen O., Thomassen M. (2000). Essential fatty acids in Atlantic salmon: time course of changes in fatty acid composition of liver, blood and carcass induced by a diet deficient in n-3 and n-6 fatty acids. *Aquaculture Nutrition*.

[10] Bell J. G., McEvoy J., Tocher D. R., McGhee F., Campbell P. J., Sargent J. R. (2001). Replacement of fish oil with rapeseed oil in diets of Atlantic salmon (*Salmo salar*) affects tissue lipid compositions and hepatocyte fatty acid metabolism. *The Journal of Nutrition*.

[11] Bell J. G., Henderson R. J., Tocher D. R. (2002). Substituting fish oil with crude palm oil in the diet of Atlantic salmon (*Salmo salar*) affects muscle fatty acid composition and hepatic fatty acid metabolism. *The Journal of Nutrition*.

[12] Bell J. G., Tocher D. R., Henderson R. J., Dick J. R., Crampton V. O. (2003). Altered fatty acid compositions in Atlantic salmon (*Salmo salar*) fed diets containing linseed and rapeseed oils can be partially restored by a subsequent fish oil finishing diet. *The Journal of Nutrition*.

[13] Rosenlund G., Obach A., Sandberg M. G., Standal H., Tveit K. (2001). Effect of alternative lipid sources on long-term growth performance and quality of Atlantic salmon (*Salmo salar* L.). *Supplement*.

[14] Torstensen B. E., Bell J. G., Rosenlund G. (2005). Tailoring of Atlantic salmon (*Salmo salar* L.) flesh lipid composition and sensory quality by replacing fish oil with a vegetable oil blend. *Journal of Agricultural and Food Chemistry*.

[15] Waagbø R., Sandnes K., Torrissen O. J., Sandvin A., Lie Ø. (1993). Chemical and sensory evaluation of fillets from Atlantic salmon (*Salmo salar*) fed three levels of N-3 polyunsaturated fatty acids at two levels of vitamin E. *Food Chemistry*.

[16] Bjerkeng B., Hatlen B., Wathne E. (1999). Deposition of astaxanthin in fillets of Atlantic salmon (*Salmo salar*) fed diets with herring, capelin, sandeel, or Peruvian high PUFA oils. *Aquaculture*.

[17] Waagbø R., Berntssen M. H. G., Danielsen T. (2013). Feeding Atlantic salmon diets with plant ingredients during the seawater phase – a full-scale net production of marine protein with focus on biological performance, welfare, product quality and safety. *Aquaculture Nutrition*.

[18] Wathne E., Bjerkeng B., Storebakken T., Vassvik V., Odland A. B. (1998). Pigmentation of Atlantic salmon (*Salmo salar*) fed astaxanthin in all meals or in alternating meals. *Aquaculture*.

[19] Ytrestøyl T., Struksnæs G., Rørvik K.-A., Bjerkeng B. Utilisation of astaxanthin in Atlantic salmon from seawater transfer to slaughter.

[20] Amengual J., Lobo G. P., Golczak M. (2011). A mitochondrial enzyme degrades carotenoids and protects against oxidative stress. *FASEB Journal*.

[21] Fakhri S., Abbaszadeh F., Dargahi L., Jorjani M. (2018). Astaxanthin: a mechanistic review on its biological activities and health benefits. *Pharmacological Research*.

[22] Guillou A., Choubert G., Storebakken T., de la Noüet J., Kaushik S. (1989). Bioconversion pathway of astaxanthin into retinol_2_ in mature rainbow trout (*Salmo Gairdneri* Rich.). *Comparative Biochemistry and Physiology*.

[23] Moren M., Naess T., Hamre K. (2002). Conversion of *β*-carotene, canthaxanthin and astaxanthin to vitamin A in Atlantic halibut (*Hippoglossus hippoglossus* L.) juveniles. *Fish Physiology and Biochemistry*.

[24] Schiedt K., Leuenberger F. J., Vecchi M., Glinz E. (1985). Absorption, retention, and metabolic transformations of carotenoids in rainbow trout, salmon and chicken. *Pure and Applied Chemistry*.

[25] Lobo G. B., Amengual J., Li H. N. M. (2010). *β*,*β*-carotene decreases peroxisome proliferator receptor *γ* activity and reduces lipid storage capacity of adipocytes in a *β*,*β*-carotene oxygenase 1-dependent manner. *Journal of Biological Chemistry*.

[26] Lobo G. P., Amengual J., Palczewski G., Babino D., von Lintig J. (2012). Mammalian carotenoid-oxygenases: key players for carotenoid function and homeostasis. *Biochimica et Biophysica Acta (BBA) - Molecular and Cell Biology of Lipids*.

[27] During A., Nagao A., Terao J. (1998). *β*-carotene 15,15′-dioxygenase activity and cellular retinol-binding protein type II level are enhanced by dietary unsaturated triacylglycerols in rat intestines. *The Journal of Nutrition*.

[28] Lietz G., Lange J., Rimbach G. (2010). Molecular and dietary regulation of *β*,*β*-carotene 15,15′-monooxygenase 1 (BCMO1). *Archives of Biochemistry and Biophysics*.

[29] Aas G. H., Bjerkeng B., Hatlen B., Storebakken T. (1997). Idoxanthin, a major carotenoid in the flesh of Arctic charr (*Salvelinus alpinus*) fed diets containing astaxanthin. *Aquaculture*.

[30] Schiedt K., Foss P., Storebakken T., Liaaen-Jensen S. (1989). Metabolism of carotenoids in salmonids--I. idoxanthin, a metabolite of astaxanthin in the flesh of atlantic salmon (*Salmon salar*, L.) under varying external conditions. *Comparative Biochemistry and Physiology*.

[31] Bjerkeng B., Hatlen B., Jobling M. (2000). Astaxanthin and its metabolites idoxanthin and crustaxanthin in flesh, skin, and gonads of sexually immature and maturing Arctic charr (*Salvelinus alpinus* (L.)). *Comparative Biochemistry and Physiology. B*.

[32] Ytrestøyl T., Coral-Hinostroza G. N., Hatlen B., Robb D. H. F., Bjerkeng B. (2004). Carotenoid and lipid content in muscle of Atlantic salmon, *Salmo salar*, transferred to seawater as 0+ or 1+ smolts. *Comparative Biochemistry and Physiology*.

[33] Bjerkeng B., Refstie S., Fjalestad K. T., Storebakken T., Rødbotten M., Roem A. (1997). Quality parameters of the flesh of Atlantic salmon (*Salmo salar*) as affected by dietary fat content and full-fat soybean meal as a partial substitute for fish meal in the diet. *Aquaculture*.

[34] Livak K. J., Schmittgen T. D. (2001). Analysis of relative gene expression data using real-time quantitative PCR and the 2^−*ΔΔC*^_T_ method. *Methods*.

[35] Borel P., Grolier P., Armand M. (1996). Carotenoids in biological emulsions: solubility, surface-to-core distribution, and release from lipid droplets. *Journal of Lipid Research*.

[36] Sigholt T., Berge G. M., Ruyter B., Åsgård T. Graded levels of palm oil in diets for Atlantic salmon (*Salmo salar*), at three different temperatures.

[37] Regost C., Jakobsen J. V., Rørå A. M. B. (2004). Flesh quality of raw and smoked fillets of Atlantic salmon as influenced by dietary oil sources and frozen storage. *Food Research International*.

[38] Rørå A. M. B., Birkeland S., Hultmann L., Rustad T., Skåra T., Bjerkeng B. (2005). Quality characteristics of farmed Atlantic salmon (*Salmo salar*) fed diets high in soybean or fish oil as affected by cold-smoking temperature. *Lebensmittelwissenschaft und Technologie – Food Science and Techhnology*.

[39] Elliott R. (2005). Mechanisms of genomic and non-genomic actions of carotenoids. *Biochimica et Biophysica Acta (BBA)-Molecular Basis of Disease*.

[40] Itoh K., Chiba T., Takahashi S. (1997). An Nrf2/small Maf heterodimer mediates the induction of phase II detoxifying enzyme genes through antioxidant response elements. *Biochemical and Biophysical Research Communications*.

[41] Motohashi H., Yamamoto M. (2004). Nrf2-Keap1 defines a physiologically important stress response mechanism. *Trends in Molecular Medicine*.

[42] De Bosscher K., Vanden Berghe W., Haegeman G. (2006). Cross-talk between nuclear receptors and nuclear factor *κ*B. *Oncogene*.

[43] Oeckinghaus A., Ghosh S. (2009). The NF-kappaB family of transcription factors and its regulation. *Cold Spring Harbor Perspectives in Biology*.

[44] Bhuvaneswari S., Arunkumar E., Viswanathan P., Anuradha C. V. (2010). Astaxanthin restricts weight gain, promotes insulin sensitivity and curtails fatty liver disease in mice fed a obesity-promoting diet. *Process Biochemistry*.

[45] Gammone M. A., Riccioni G., D'Orazio N. (2015). Carotenoids: potential allies of cardiovascular health?. *Food & Nutrition Research*.

[46] Helgeland H., Sodeland M., Zoric N. (2019). Genomic and functional gene studies suggest a key role of *beta-carotene oxygenase 1 like (bco1l)* gene in salmon flesh color. *Scientific Reports*.

[47] Boulanger A., McLemore P., Copeland N. G. (2003). Identification of beta-carotene 15,15′-monooxygenase as a peroxisome proliferator-activated receptor target gene. *The FASEB Journal*.

[48] Raju M., Lakshminarayana R., Krishnakantha T. P., Baskaran V. (2006). Micellar oleic and eicosapentaenoic acid but not linoleic acid influences the *β*-carotene uptake and its cleavage into retinol in rats. *Molecular and Cellular Biochemistry*.

