# Supplementary material for: Dietary Level of the Omega-3 Fatty Acids EPA and DHA Influence the Flesh Pigmentation in Atlantic Salmon
Source: Aquac Nutr. 2023 Mar 2;2023:5528942. doi: 10.1155/2023/5528942 (PMC9998164; doi:10.1155/2023/5528942)
Supplement: Supplementary Materials — The tables in the supplemental files show the muscle fatty acid composition at 1.2 (Table S1) and 3.5 kg (Table S2) in all dietary treatments. Figure S1 shows the effect of diet in Period 1 (50-400 g) and Period 2 (400-3500 g) on the expression of the cox2 gene when the fish was 3.5 kg. [file 5528942.f1.docx]

**Supplements**

Table S1a. Pre-diet effects in the 0.2% dietary group at 1.2 kg:

Fatty acid composition (% of total) in the muscle of Atlantic salmon fed 0%, 1% and 2.0% EPA and DHA in the experimental diets in Period 1 (50-400 g) and 0.2% EPA+DHA from 400 g to 1.2 kg (Period 2A). Data are shown as mean ± SEM (n = 3, being each sample represented by a pool of 2 fish)

| Period 2A | **0.2% EPA+DHA** | | | | | | | | |
| --- | --- | --- | --- | --- | --- | --- | --- | --- | --- |
| Period 1 |  | EPA | |  | DHA | |  | EPA+DHA | |
|  | 0 % | 1.0% | 2.0% |  | 1.0% | 2.0% |  | 1.0% | 2.0% |
| 14:0 | 0.8 ± 0.01 | 0.8 ± 0.01 | 0.8 ± 0.00 |  | 0.8 ± 0.01 | 0.8 ± 0.00 |  | 0.8 ± 0.02 | 0.8 ± 0.01 |
| 16:0 | 13.2 ± 0.09 | 13.4 ± 0.05 | 13.3 ± 0.09 | | 13.5 ± 0.04 | 8.7 ± 4.36 |  | 8.7 ± 4.37 | 13.6 ± 0.12 |
| 18:0 | 4.5 ± 0.06 | 4.4 ± 0.05 | 4.3 ± 0.07 |  | 4.4 ± 0.02 | 4.4 ± 0.05 |  | 4.3 ± 0.09 | 4.5 ± 0.04 |
| Σ n-0 | 19.9 ± 0.06 | 19.8 ± 0.12 | 19.6 ± 0.14 | | 19.9 ± 0.09 | 15.0 ± 4.27 | | 15.0 ± 4.38 | 19.9 ± 0.11 |
| 16:1 n-7 | 2.2 ± 0.01 | 2.2 ± 0.01 | 2.2 ± 0.01 |  | 2.2 ± 0.03 | 2.3 ± 0.02 |  | 2.2 ± 0.03 | 2.3 ± 0.02 |
| 18:1 n-9 | 36.9 ± 0.67 | 37.2 ± 0.18 | 36.9 ± 0.08 | | 37.5 ± 0.28 | 37.4 ± 0.34 | | 37.4 ± 0.28 | 37.5 ± 0.17 |
| 20:1 n-9 | 1.3 ± 0.02 | 1.3 ± 0.04 | 1.3 ± 0.03 |  | 1.3 ± 0.01 | 1.3 ± 0.03 |  | 1.3 ± 0.02 | 1.3 ± 0.03 |
| 22:1 n-9 | 0.2 ± 0.01 | 0.2 ± 0.00 | 0.2 ± 0.01 |  | 0.2 ± 0.00 | 0.2 ± 0.01 |  | 0.2 ± 0.00 | 0.2 ± 0.01 |
| Σ MUFA | 43.7 ± 0.11 | 43.4 ± 0.15 | 43.2 ± 0.12 | | 43.7 ± 0.40 | 48.4 ± 4.39 | | 48.0 ± 4.33 | 43.8 ± 0.06 |
| 18:2 n-6 | 17.6 ± 0.17 | 17.9 ± 0.04 | 17.9 ± 0.02 | | 18.0 ± 0.07 | 17.9 ± 0.11 | | 18.3 ± 0.20 | 17.8 ± 0.09 |
| 18:3 n-6 | 1.0 ± 0.05 | 0.8 ± 0.06 | 0.8 ± 0.02 |  | 0.8 ± 0.04 | 0.8 ± 0.03 |  | 0.8 ± 0.02 | 0.7 ± 0.04 |
| 20:4 n-6 | 1.3 ± 0.08 | 1.2 ± 0.05 | 1.1 ± 0.04 |  | 1.1 ± 0.01 | 1.1 ± 0.02 |  | 1.1 ± 0.03 | 1.1 ± 0.07 |
| Σ n-6 | 20.9 ± 0.16 | 20.9 ± 0.04 | 20.8 ± 0.04 | | 21.0 ± 0.08 | 20.9 ± 0.11 | | 21.2 ± 0.18 | 20.7 ± 0.09 |
| 18:3 n-3 | 7.6 ± 0.09 | 7.5 ± 0.12 | 7.7 ± 0.08 |  | 7.7 ± 0.08 | 7.7 ± 0.10 |  | 7.9 ± 0.17 | 7.7 ± 0.08 |
| 20:5 n-3 | 1.1 ± 0.03 | 1.4 ± 0.07 | 1.7 ± 0.09 |  | 1.0 ± 0.04 | 1.0 ± 0.03 |  | 1.2 ± 0.10 | 1.2 ± 0.01 |
| 22:5 n-3 | 0.4 ± 0.01 | 0.5 ± 0.02 | 0.5 ± 0.02 |  | 0.4 ± 0.01 | 0.4 ± 0.01 |  | 0.4 ± 0.04 | 0.4 ± 0.00 |
| 22:6 n-3 | 2.0 ± 0.14 | 2.6 ± 0.12 | 2.6 ± 0.12 |  | 2.7 ± 0.20 | 2.8 ± 0.19 |  | 2.7 ± 0.24 | 2.6 ± 0.10 |
| Σ n-3 | 11.1 ± 0.24 | 12.1 ± 0.21 | 12.6 ± 0.17 | | 11.8 ± 0.25 | 12.0 ± 0.14 | | 12.3 ± 0.21 | 12.0 ± 0.07 |
| EPA+DHA | 3.1 ± 0.16 | 4.1 ± 0.11 | 4.3 ± 0.20 |  | 3.7 ± 0.20 | 3.8 ± 0.22 |  | 3.9 ± 0.33 | 3.8 ± 0.10 |

Table S1b. Pre-diet effects in the 1% dietary group at 1.2 kg:

Fatty acid composition (% of total) in the muscle of Atlantic salmon fed 0%, 1% and 2% EPA and DHA in the experimental diets in Period 1 (50-400 g) and 1% EPA+DHA from 400 g to 1.2 kg (Period 2A). Data are shown as mean ± SEM (n = 3, being each sample represented by a pool of 2 fish)

| Period 2A | **1.0% EPA+DHA** | | | | | | | | | |
| --- | --- | --- | --- | --- | --- | --- | --- | --- | --- | --- |
| Period 1 |  | EPA | | |  | DHA | |  | EPA+DHA | |
|  | 0 % | 1.0% | 2.0% | |  | 1.0% | 2.0% |  | 1.0% | 2.0% |
| 14:0 | 1.9 ± 0.06 | 1.9 ± 0.02 | 1.9 ± 0.07 | |  | 1.9 ± 0.04 | 1.9 ± 0.03 |  | 1.9 ± 0.04 | 1.9 ± 0.02 |
| 16:0 | 13.2 ± 0.23 | 13.3 ± 0.04 | 13.4 ± 0.03 | 13.4 ± 0.19 | | | 12.9 ± 0.20 | | 13.4 ± 0.16 | 13.1 ± 0.10 |
| 18:0 | 4.0 ± 0.03 | 4.0 ± 0.03 | 4.1 ± 0.03 | |  | 4.1 ± 0.09 | 4.0 ± 0.11 |  | 4.0 ± 0.07 | 3.9 ± 0.03 |
| Σ n-0 | 20.3 ± 0.28 | 20.2 ± 0.01 | 20.3 ± 0.05 | 20.4 ± 0.12 | | | 19.8 ± 0.28 | | 20.4 ± 0.23 | 20.0 ± 0.13 |
| 16:1 n-7 | 3.5 ± 0.13 | 3.4 ± 0.07 | 3.4 ± 0.09 | |  | 3.3 ± 0.08 | 3.5 ± 0.17 |  | 3.4 ± 0.07 | 3.4 ± 0.10 |
| 18:1 n-9 | 32.1 ± 0.78 | 32.4 ± 0.87 | 31.9 ± 1.13 | 32.0 ± 0.86 | | | 32.2 ± 0.77 | | 32.1 ± 0.88 | 32.0 ± 1.10 |
| 20:1 n-9 | 2.4 ± 1.09 | 3.4 ± 0.09 | 3.2 ± 0.24 | |  | 3.4 ± 0.19 | 3.6 ± 0.12 |  | 3.5 ± 0.10 | 3.4 ± 0.09 |
| 22:1 n-9 | 0.5 ± 0.02 | 0.5 ± 0.02 | 0.5 ± 0.05 | |  | 0.5 ± 0.03 | 0.5 ± 0.02 |  | 0.5 ± 0.02 | 0.5 ± 0.03 |
| Σ MUFA | 40.9 ± 1.38 | 41.9 ± 0.86 | 41.2 ± 0.93 | 41.4 ± 0.86 | | | 41.9 ± 0.96 | | 41.9 ± 0.77 | 41.6 ± 1.17 |
| 18:2 n-6 | 14.4 ± 0.25 | 14.9 ± 0.16 | 14.8 ± 0.27 | 14.7 ± 0.43 | | | 14.6 ± 0.05 | | 14.6 ± 0.30 | 14.7 ± 0.08 |
| 18:3 n-6 | 0.4 ± 0.04 | 0.4 ± 0.00 | 0.3 ± 0.01 | |  | 0.3 ± 0.11 | 0.2 ± 0.08 |  | 0.3 ± 0.12 | 0.4 ± 0.04 |
| 20:4 n-6 | 0.7 ± 0.07 | 0.6 ± 0.01 | 0.6 ± 0.04 | |  | 0.6 ± 0.05 | 0.6 ± 0.01 |  | 0.7 ± 0.02 | 0.7 ± 0.03 |
| Σ n-6 | 16.4 ± 0.17 | 16.9 ± 0.15 | 16.7 ± 0.27 | 16.6 ± 0.35 | | | 16.6 ± 0.01 | | 16.6 ± 0.23 | 16.8 ± 0.13 |
| 18:3 n-3 | 8.4 ± 0.00 | 8.5 ± 0.10 | 8.6 ± 0.11 | |  | 8.6 ± 0.28 | 8.6 ± 0.02 |  | 8.3 ± 0.18 | 8.5 ± 0.01 |
| 20:5 n-3 | 1.8 ± 0.05 | 1.9 ± 0.04 | 2.4 ± 0.04 | |  | 1.8 ± 0.07 | 1.6 ± 0.02 |  | 1.8 ± 0.06 | 1.9 ± 0.12 |
| 22:5 n-3 | 0.6 ± 0.01 | 0.6 ± 0.02 | 0.8 ± 0.01 | |  | 0.6 ± 0.03 | 0.6 ± 0.02 |  | 0.6 ± 0.03 | 0.6 ± 0.02 |
| 22:6 n-3 | 3.7 ± 0.32 | 3.5 ± 0.03 | 3.9 ± 0.13 | |  | 4.0 ± 0.07 | 4.2 ± 0.12 |  | 3.9 ± 0.05 | 4.1 ± 0.34 |
| Σ n-3 | 14.9 ± 0.34 | 14.8 ± 0.12 | 15.7 ± 0.31 | 15.2 ± 0.24 | | | 15.2 ± 0.08 | | 14.8 ± 0.07 | 15.4 ± 0.47 |
| EPA+DHA | 5.5 ± 0.37 | 5.4 ± 0.07 | 6.2 ± 0.17 | |  | 5.8 ± 0.11 | 5.8 ± 0.14 |  | 5.7 ± 0.06 | 6.0 ± 0.46 |

Table S1c. Pre-diet effects in the 1.7% dietary group at 1.2 kg:

Fatty acid composition (% of total) in the muscle of Atlantic salmon fed 0%, 1%, 2% EPA and DHA in the experimental diets in Period 1 (50-400 g) and 1.7% EPA+DHA from 400 g to 1.2 kg (Period 2A). Data are shown as mean ± SEM (n = 3, being each sample represented by a pool of 2 fish)

| Period 2A | **1.7% EPA+DHA** | | | | | | | | |
| --- | --- | --- | --- | --- | --- | --- | --- | --- | --- |
| Period 1 |  | EPA | |  | DHA | |  | EPA+DHA | |
|  | 0 % | 1.0% | 2.0% |  | 1.0% | 2.0% |  | 1.0% | 2.0% |
| 14:0 | 3.0 ± 0.20 | 2.8 ± 0.13 | 2.7 ± 0.17 |  | 3.0 ± 0.02 | 2.9 ± 0.10 |  | 2.9 ± 0.04 | 2.9 ± 0.14 |
| 16:0 | 11.1 ± 0.13 | 10.9 ± 0.13 | 11.1 ± 0.05 | 11.1 ± 0.12 | | 11.1 ± 0.06 | | 11.0 ± 0.04 | 11.0 ± 0.04 |
| 18:0 | 2.7 ± 0.06 | 2.7 ± 0.07 | 2.8 ± 0.02 |  | 2.6 ± 0.01 | 2.8 ± 0.05 |  | 2.7 ± 0.06 | 2.7 ± 0.04 |
| Σ n-0 | 17.7 ± 0.23 | 17.4 ± 0.27 | 17.5 ± 0.13 | 17.7 ± 0.16 | | 17.7 ± 0.05 | | 17.5 ± 0.01 | 17.5 ± 0.15 |
| 16:1 n-7 | 4.2 ± 0.28 | 4.0 ± 0.22 | 3.7 ± 0.20 |  | 4.2 ± 0.12 | 4.1 ± 0.06 |  | 4.0 ± 0.09 | 4.1 ± 0.06 |
| 18:1 n-9 | 33.2 ± 1.50 | 34.0 ± 1.03 | 34.4 ± 1.43 | 32.3 ± 1.42 | | 33.3 ± 1.28 | | 33.9 ± 1.07 | 33.3 ± 1.40 |
| 20:1 n-9 | 7.0 ± 0.54 | 6.7 ± 0.43 | 6.2 ± 0.62 |  | 7.1 ± 0.30 | 7.0 ± 0.38 |  | 6.9 ± 0.24 | 6.9 ± 0.38 |
| 22:1 n-9 | 4.4 ± 1.83 | 3.9 ± 1.60 | 3.7 ± 1.56 |  | 4.2 ± 1.69 | 4.2 ± 1.72 |  | 4.0 ± 1.61 | 4.3 ± 1.75 |
| Σ MUFA | 50.8 ± 0.95 | 50.5 ± 0.78 | 49.8 ± 0.97 | 49.7 ± 0.79 | | 50.4 ± 0.89 | | 50.8 ± 0.69 | 50.5 ± 0.82 |
| 18:2 n-6 | 11.0 ± 0.39 | 11.5 ± 0.35 | 11.8 ± 0.37 | 11.2 ± 0.05 | | 11.2 ± 0.24 | | 11.4 ± 0.08 | 11.3 ± 0.18 |
| 18:3 n-6 | 0.3 ± 0.02 | 0.3 ± 0.01 | 0.3 ± 0.02 |  | 0.3 ± 0.01 | 0.2 ± 0.01 |  | 0.3 ± 0.03 | 0.3 ± 0.01 |
| 20:4 n-6 | 0.5 ± 0.04 | 0.4 ± 0.01 | 0.4 ± 0.03 |  | 0.4 ± 0.01 | 0.4 ± 0.01 |  | 0.4 ± 0.05 | 0.4 ± 0.02 |
| Σ n-6 | 12.7 ± 0.50 | 13.1 ± 0.40 | 13.5 ± 0.39 | 12.8 ± 0.02 | | 12.8 ± 0.22 | | 13.1 ± 0.09 | 12.9 ± 0.22 |
| 18:3 n-3 | 3.6 ± 0.13 | 3.8 ± 0.15 | 3.9 ± 0.08 |  | 3.7 ± 0.06 | 3.7 ± 0.04 |  | 3.8 ± 0.04 | 3.7 ± 0.04 |
| 20:5 n-3 | 2.6 ± 0.09 | 2.7 ± 0.05 | 2.9 ± 0.07 |  | 2.5 ± 0.07 | 2.5 ± 0.06 |  | 2.5 ± 0.05 | 2.8 ± 0.03 |
| 22:5 n-3 | 0.9 ± 0.01 | 1.0 ± 0.00 | 1.0 ± 0.01 |  | 0.9 ± 0.03 | 0.9 ± 0.03 |  | 0.9 ± 0.04 | 1.0 ± 0.01 |
| 22:6 n-3 | 4.1 ± 0.14 | 4.2 ± 0.17 | 4.7 ± 0.48 |  | 4.7 ± 0.16 | 4.7 ± 0.20 |  | 4.1 ± 0.09 | 4.6 ± 0.36 |
| Σ n-3 | 11.4 ± 0.27 | 11.9 ± 0.43 | 12.7 ± 0.73 | 12.0 ± 0.36 | | 12.0 ± 0.32 | | 11.5 ± 0.10 | 12.2 ± 0.42 |
| EPA+DHA | 6.6 ± 0.09 | 6.9 ± 0.21 | 7.6 ± 0.55 |  | 7.2 ± 0.24 | 7.2 ± 0.22 |  | 6.6 ± 0.05 | 7.4 ± 0.34 |

Table S1d. P-values for effects of diet in Period 1 and 2A on fillet fatty acid composition at 1.2 kg.

|  | Period 2A | Period 1 | Interaction |
| --- | --- | --- | --- |
| 14:0 | <0.0001 | 0.0114 | ns |
| 16:0 | 0.0183 | ns | ns |
| 18:0 | <0.0001 | ns | 0.0423 |
| Σ n-0 | 0.0032 | ns | ns |
| 16:1 n-7 | <0.0001 | ns | ns |
| 18:1 n-9 | <0.0001 | ns | ns |
| 20:1 n-9 | <0.0001 | ns | ns |
| 22:1 n-9 | <0.0001 | ns | ns |
| Σ MUFA | <0.0001 | ns | ns |
| 18:2 n-6 | <0.0001 | 0.058 | ns |
| 18:3 n-6 | <0.0001 | 0.021 | ns |
| 20:4 n-6 | <0.0001 | 0.0041 | ns |
| Σ n-6 | <0.0001 | 0.0161 | ns |
| 18:3 n-3 | <0.0001 | ns | ns |
| 20:5 n-3 | <0.0001 | <0.0001 | ns |
| 22:5 n-3 | <0.0001 | <0.0001 | ns |
| 22:6 n-3 | <0.0001 | 0.0062 | ns |
| Σ n-3 | <0.0001 | 0.0035 | ns |
| EPA+DHA | <0.0001 | 0.0013 | ns |

Table S2a. Pre-diet effects in the 0.2% dietary group at 3.5 kg:

Fatty acid composition (% of total) in the muscle of Atlantic salmon at 3.5 kg in the 0.2% group that was fed 0%, 1% and 2% EPA and DHA in the experimental diets in Period 1 (50-400 g) and 0.2% EPA+DHA from 400 g to 3.5 kg (Period 2A and B). Data are shown as mean ± SEM (n = 3, being each sample represented by a pool of 2 fish)

| Diet Period 2B | 02% EPA+DHA | | | |
| --- | --- | --- | --- | --- |
| Diet Period 1 | 0% | 2.0% EPA | 2.0%DHA | 2.0% EPA+DHA |
| Fat (%) | 15.0 ± 1.08 | 15.8 ± 0.34 | 16.7 ± 0.35 | 12.9 ± 0.31 |
| 14:0 | 1.1 ± 0.02 | 1.1 ± 0.01 | 1.1 ± 0.02 | 1.1 ± 0.08 |
| 16:0 | 12.6 ± 0.18 | 12.6 ± 0.27 | 12.7 ± 0.11 | 12.6 ± 0.29 |
| 18:0 | 4.2 ± 0.02 | 4.2 ± 0.07 | 4.2 ± 0.03 | 4.2 ± 0.20 |
| Σ n-0 | 1.5 ± 0.01 | 1.5 ± 0.01 | 1.5 ± 0.02 | 1.5 ± 0.05 |
| 16:1 n-7 | 2.6 ± 0.02 | 3.0 ± 0.05 | 2.6 ± 0.02 | 2.7 ± 0.14 |
| 18:1 n-9 | 37.8 ± 0.14 | 38.0 ± 0.20 | 37.7 ± 0.03 | 37.6 ± 0.37 |
| 18:1 n-7 | 1.8 ± 0.07 | 1.5 ± 0.18 | 1.9 ± 0.02 | 1.7 ± 0.21 |
| 20:1 n-9 | 1.8 ± 0.08 | 1.8 ± 0.04 | 1.8 ± 0.03 | 1.7 ± 0.25 |
| 20:1 n-11 | 1.4 ± 0.09 | 1.5 ± 0.11 | 1.5 ± 0.07 | 1.7 ± 0.09 |
| 22:1 n-7 | 0.7 ± 0.02 | 0.6 ± 0.10 | 0.7 ± 0.01 | 0.7 ± 0.07 |
| 22:1 n-11 | 0.7 ± 0.06 | 0.7 ± 0.01 | 0.6 ± 0.09 | 0.7 ± 0.12 |
| 22:1 n-9 | 0.2 ± 0.01 | 0.2 ± 0.00 | 0.2 ± 0.00 | 0.2 ± 0.00 |
| Σ MUFA | 48.0 ± 0.05 | 48.0 ± 0.17 | 47.9 ± 0.05 | 47.6 ± 0.08 |
| 18:2 n-6 | 17.9 ± 0.11 | 18.0 ± 0.16 | 18.1 ± 0.07 | 17.7 ± 0.17 |
| 18:3 n-6 | 0.7 ± 0.04 | 0.7 ± 0.06 | 0.7 ± 0.04 | 0.8 ± 0.06 |
| 18:3 n-3 | 8.1 ± 0.11 | 8.2 ± 0.05 | 8.1 ± 0.09 | 7.8 ± 0.25 |
| 20:2 n-6 | 0.9 ± 0.07 | 0.9 ± 0.03 | 1.0 ± 0.02 | 0.9 ± 0.07 |
| 20:3 n-6 | 0.9 ± 0.02 | 0.8 ± 0.03 | 0.8 ± 0.03 | 1.0 ± 0.14 |
| 20:4 n-6 | 0.5 ± 0.01 | 0.4 ± 0.01 | 0.4 ± 0.02 | 0.5 ± 0.03 |
| 20:3 n-3 | 0.4 ± 0.03 | 0.4 ± 0.02 | 0.4 ± 0.02 | 0.3 ± 0.04 |
| 20:5 n-3 | 1.2 ± 0.06 | 1.5 ± 0.06 | 1.2 ± 0.02 | 1.6 ± 0.28 |
| 22:5 n-3 | 0.4 ± 0.01 | 0.4 ± 0.04 | 0.4 ± 0.03 | 0.5 ± 0.06 |
| 22:6 n-3 | 1.3 ± 0.08 | 1.3 ± 0.06 | 1.5 ± 0.07 | 1.6 ± 0.10 |
| Σ PUFA | 32.7 ± 0.26 | 32.8 ± 0.33 | 32.8 ± 0.09 | 32.8 ± 0.29 |
| n3/n6 | 0.2 ± 0.01 | 0.2 ± 0.01 | 0.2 ± 0.00 | 0.2 ± 0.01 |

Table S2b. Pre-diet effects in the 1% dietary group at 3.5 kg:

Fatty acid composition (% of total) in the muscle of Atlantic salmon at 3.5 kg in the 1% group that was fed 0%, 1% and 2% EPA and DHA in the experimental diets in Period 1 (50-400 g) and 1% EPA+DHA from 400 g to 3.5 kg (Period 2A and B). Data are shown as mean ± SEM (n = 3, being each sample represented by a pool of 2 fish)

| Diet Period 2B | 1.0% EPA+DHA | | | |
| --- | --- | --- | --- | --- |
| Diet Period 1 | 0%EPA+DHA | 2.0% EPA | 2.0%DHA | 2.0%EPA+DHA |
| Fat (%) | 17.1 ± 0.89 | 16.8 ± 1.23 | 16.7 ± 1.05 | 13.9 ± 2.76 |
| 14:0 | 2.6 ± 0.03 | 2.6 ± 0.02 | 2.5 ± 0.13 | 2.4 ± 0.19 |
| 16:0 | 11.8 ± 0.05 | 12.0 ± 0.17 | 11.7 ± 0.14 | 11.4 ± 0.24 |
| 18:0 | 3.3 ± 0.04 | 3.4 ± 0.04 | 3.4 ± 0.05 | 3.5 ± 0.11 |
| Σ n-0 | 3.0 ± 0.04 | 2.9 ± 0.07 | 2.9 ± 0.11 | 2.6 ± 0.26 |
| 16:1 n-7 | 4.1 ± 0.06 | 4.2 ± 0.02 | 4.1 ± 0.15 | 3.8 ± 0.04 |
| 18:1 n-9 | 29.7 ± 0.19 | 30.3 ± 0.41 | 30.4 ± 0.77 | 30.7 ± 0.47 |
| 18:1 n-7 | 2.3 ± 0.04 | 2.1 ± 0.15 | 2.3 ± 0.03 | 2.1 ± 0.19 |
| 20:1 n-9 | 5.7 ± 0.01 | 5.6 ± 0.07 | 5.4 ± 0.26 | 5.3 ± 0.21 |
| 20:1 n-11 | 1.1 ± 0.03 | 1.2 ± 0.07 | 1.2 ± 0.01 | 1.0 ± 0.03 |
| 22:1 n-7 | 0.8 ± 0.02 | 0.9 ± 0.03 | 0.8 ± 0.03 | 0.8 ± 0.05 |
| 22:1 n-11 | 5.2 ± 0.07 | 5.1 ± 0.06 | 4.8 ± 0.38 | 4.4 ± 0.30 |
| 22:1 n-9 | 0.7 ± 0.09 | 1.0 ± 0.22 | 0.8 ± 0.08 | 0.6 ± 0.00 |
| Σ MUFA | 51.2 ± 0.22 | 51.6 ± 0.32 | 51.1 ± 0.31 | 49.8 ± 0.18 |
| 18:2 n-6 | 13.0 ± 0.09 | 13.0 ± 0.22 | 13.3 ± 0.39 | 13.9 ± 0.37 |
| 18:3 n-6 | 0.3 ± 0.01 | 0.4 ± 0.02 | 0.4 ± 0.02 | 0.3 ± 0.01 |
| 18:3 n-3 | 8.3 ± 0.03 | 7.9 ± 0.10 | 8.1 ± 0.07 | 8.1 ± 0.02 |
| 20:2 n-6 | 0.8 ± 0.03 | 0.8 ± 0.05 | 0.9 ± 0.05 | 0.9 ± 0.04 |
| 20:3 n-6 | 0.5 ± 0.01 | 0.5 ± 0.02 | 0.5 ± 0.05 | 0.5 ± 0.02 |
| 20:4 n-6 | 0.3 ± 0.01 | 0.3 ± 0.02 | 0.3 ± 0.02 | 0.3 ± 0.02 |
| 20:3 n-3 | 0.4 ± 0.03 | 0.4 ± 0.05 | 0.4 ± 0.02 | 0.4 ± 0.04 |
| 20:5 n-3 | 2.0 ± 0.04 | 1.8 ± 0.24 | 2.1 ± 0.11 | 2.5 ± 0.39 |
| 22:5 n-3 | 0.6 ± 0.08 | 0.7 ± 0.04 | 0.7 ± 0.03 | 0.7 ± 0.03 |
| 22:6 n-3 | 2.1 ± 0.03 | 2.4 ± 0.10 | 2.3 ± 0.03 | 2.9 ± 0.49 |
| Σ PUFA | 29.3 ± 0.07 | 28.6 ± 0.07 | 29.7 ± 0.48 | 31.1 ± 0.74 |
| n3/n6 | 0.4 ± 0.00 | 0.3 ± 0.01 | 0.4 ± 0.02 | 0.4 ± 0.01 |

Table S2c. Pre-diet effects in the 1.7% dietary group at 3.5 kg:

Fatty acid composition (% of total) in the muscle of Atlantic salmon at 3.5 kg in the 1.7% group that was fed 0%, 1% and 2% EPA and DHA in the experimental diets in Period 1 (50-400 g) and 1.7% EPA+DHA from 400 g to 3.5 kg (Period 2A and B). Data are shown as mean ± SEM (n = 3, being each sample represented by a pool of 2 fish)

| Diet Period 2B | 1.7% EPA+DHA | | | |
| --- | --- | --- | --- | --- |
| Diet Period 1 | 0%EPA+DHA | 2.0% EPA | 2.0% DHA | 2.0% EPA+DHA |
| Fat (%) | 17.0 ± 1.05 | 17.6 ± 1.36 | 18.7 ± 1.78 | 18.8 ± 1.22 |
| 14:0 | 3.8 ± 0.04 | 3.8 ± 0.03 | 3.8 ± 0.06 | 3.7 ± 0.07 |
| 16:0 | 10.0 ± 0.11 | 9.9 ± 0.06 | 9.9 ± 0.08 | 9.9 ± 0.05 |
| 18:0 | 2.2 ± 0.03 | 2.2 ± 0.01 | 2.1 ± 0.04 | 2.2 ± 0.02 |
| Σ n-0 | 4.0 ± 0.04 | 4.1 ± 0.06 | 4.1 ± 0.02 | 4.0 ± 0.06 |
| 16:1 n-7 | 4.9 ± 0.01 | 4.9 ± 0.04 | 4.8 ± 0.08 | 4.8 ± 0.02 |
| 18:1 n-9 | 29.4 ± 0.25 | 29.6 ± 0.28 | 29.7 ± 0.09 | 29.6 ± 0.20 |
| 18:1 n-7 | 3.0 ± 0.05 | 2.5 ± 0.25 | 2.4 ± 0.22 | 2.6 ± 0.17 |
| 20:1 n-9 | 9.1 ± 0.07 | 9.3 ± 0.16 | 9.5 ± 0.34 | 9.4 ± 0.21 |
| 20:1 n-11 | 0.7 ± 0.11 | 0.9 ± 0.01 | 0.8 ± 0.01 | 0.8 ± 0.02 |
| 22:1 n-7 | 0.7 ± 0.01 | 0.7 ± 0.01 | 0.8 ± 0.03 | 0.8 ± 0.03 |
| 22:1 n-11 | 8.6 ± 0.15 | 9.6 ± 0.43 | 10.5 ± 0.39 | 9.1 ± 0.40 |
| 22:1 n-9 | 1.7 ± 0.21 | 0.5 ± 0.50 | 0.0 ± 0.00 | 0.6 ± 0.61 |
| Σ MUFA | 60.0 ± 0.18 | 59.8 ± 0.15 | 60.4 ± 0.61 | 59.4 ± 0.37 |
| 18:2 n-6 | 9.6 ± 0.04 | 9.7 ± 0.07 | 9.8 ± 0.13 | 9.8 ± 0.07 |
| 18:3 n-6 | 0.3 ± 0.01 | 0.2 ± 0.04 | 0.2 ± 0.01 | 0.2 ± 0.00 |
| 18:3 n-3 | 3.4 ± 0.03 | 3.5 ± 0.02 | 3.5 ± 0.04 | 3.5 ± 0.04 |
| 20:2 n-6 | 0.4 ± 0.21 | 0.7 ± 0.01 | 0.8 ± 0.01 | 0.8 ± 0.01 |
| 20:3 n-6 | 0.4 ± 0.02 | 0.4 ± 0.01 | 0.3 ± 0.03 | 0.4 ± 0.02 |
| 20:4 n-6 | 0.2 ± 0.02 | 0.2 ± 0.00 | 0.2 ± 0.02 | 0.2 ± 0.01 |
| 20:3 n-3 | 0.2 ± 0.01 | 0.2 ± 0.01 | 0.3 ± 0.00 | 0.3 ± 0.01 |
| 20:5 n-3 | 2.3 ± 0.27 | 2.7 ± 0.06 | 2.2 ± 0.15 | 2.8 ± 0.48 |
| 22:5 n-3 | 1.0 ± 0.01 | 1.0 ± 0.01 | 1.0 ± 0.01 | 1.0 ± 0.01 |
| 22:6 n-3 | 3.0 ± 0.03 | 2.9 ± 0.06 | 2.9 ± 0.23 | 3.0 ± 0.13 |
| Σ PUFA | 21.2 ± 0.53 | 22.1 ± 0.30 | 21.6 ± 0.52 | 22.2 ± 0.33 |
| n3/n6 | 0.7 ± 0.04 | 0.6 ± 0.01 | 0.6 ± 0.02 | 0.6 ± 0.04 |

Table S2d. P-values for effects of diet in Period 1 and 2 on fillet fatty acid composition at 3.5 kg.

|  | Diet Period 2 | Diet Period 1 | Interaction |
| --- | --- | --- | --- |
| 14:0 | <.0001 | ns | ns |
| 16:0 | <.0001 | ns | ns |
| 18:0 | <.0001 | ns | ns |
| Σ n-0 | <.0001 | ns | ns |
| 16:1 n-7 | <.0001 | 0.0003 | 0.03 |
| 18:1 n-9 | <.0001 | ns | ns |
| 18:1 n-7 | <.0001 | ns | ns |
| 20:1 n-9 | <.0001 | ns | ns |
| 20:1 n-11 | <.0001 | ns | ns |
| 22:1 n-7 | <.0001 | ns | ns |
| 22:1 n-11 | <.0001 | 0.05 | 0.003 |
| 22:1 n-9 | ns | ns | ns |
| Σ MUFA | <.0001 | 0.003 | ns |
| 18:2 n-6 | <.0001 | ns | ns |
| 18:3 n-6 | <.0001 | ns | ns |
| 18:3 n-3 | <.0001 | ns | 0.004 |
| 20:2 n-6 | <.0001 | ns | ns |
| 20:3 n-6 | <.0001 | ns | ns |
| 20:4 n-6 | <.0001 | ns | ns |
| 20:5 n-3 | <.0001 | 0.04 | ns |
| 22:5 n-3 | <.0001 | 0.05 | ns |
| 22:6 n-3 | <.0001 | 0.02 | ns |
| Σ PUFA | <.0001 | 0.01 | 0.03 |
| n3/n6 | <.0001 | 0.04 | 0.009 |

Figure S1.


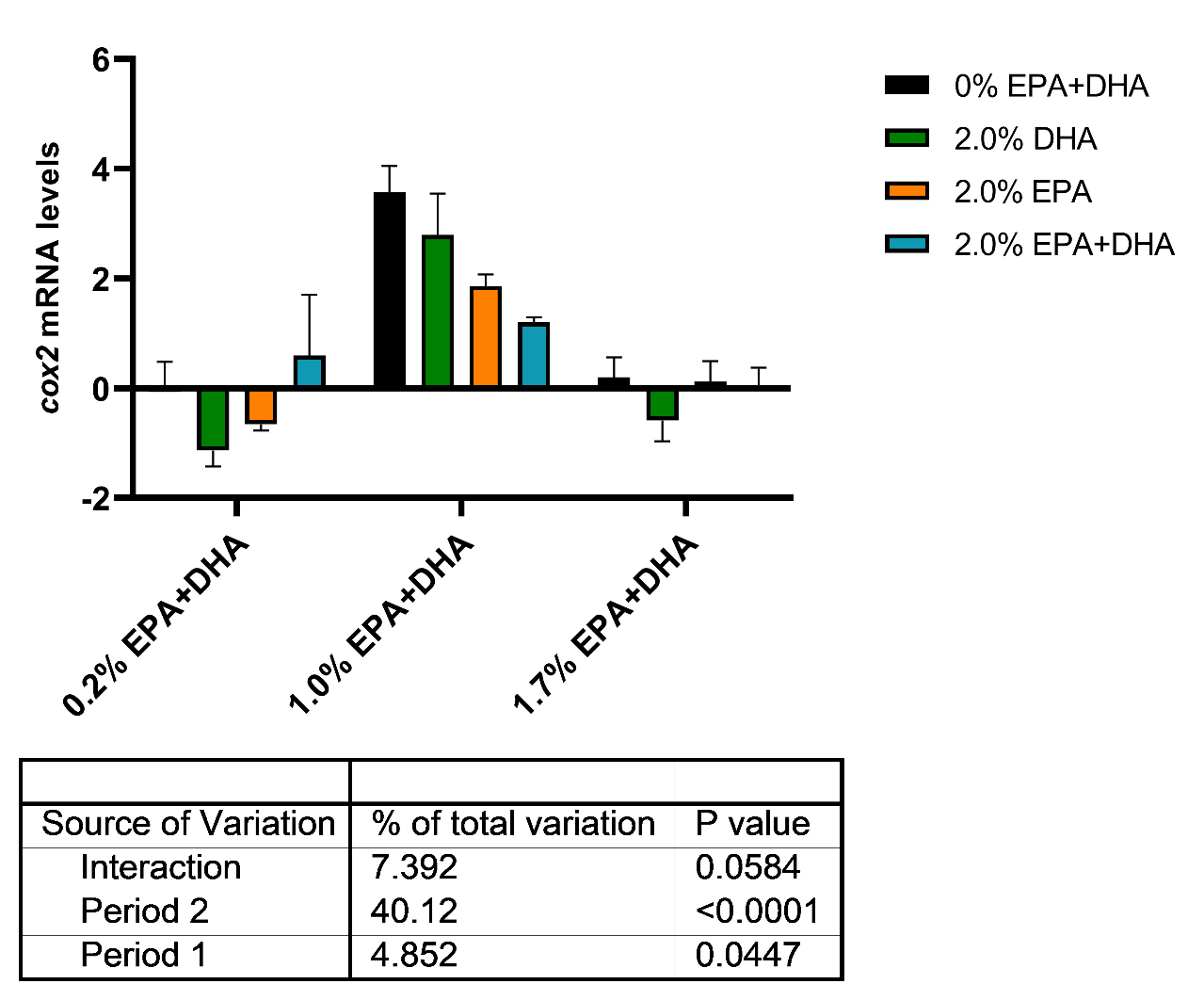


Figure S1: Effect of the diet in Period 1 (50-400 g) and Period 2 (400-3500 g) on the expression of the *cox2* gene when the fish was 3.5 kg. Values are mean+SEM
